# System for automatic heart rate calculation in epileptic seizures

**DOI:** 10.1007/s13246-017-0557-z

**Published:** 2017-05-18

**Authors:** Marcin Kołodziej, Andrzej Majkowski, Remigiusz J. Rak, Bartosz Świderski, Andrzej Rysz

**Affiliations:** 1 Institute of the Theory of Electrical Engineering, Measurements and Information Systems, Warsaw University of Technology, Koszykowa 75, 00-662 Warsaw, Poland; 2 Warsaw University of Life Sciences, Warsaw, Poland; 3 Department of Neurosurgery, Medical University of Warsaw, Warsaw, Poland

**Keywords:** Epilepsy, Heart rate, HR, Heart rate variability, R-wave, RR interval, QRS detection

## Abstract

This article presents a comprehensive system for automatic heart rate (HR) detection. The system is robust and resistant to disturbances (noise, interferences, artifacts) occurring mainly during epileptic seizures. ECG signal filtration (IIR) and normalization due to skewness and standard deviation were used as preprocessing steps. A key element of the system is a reference QRS complex pattern calculated individually for each ECG recording. Next, a cross-correlation of the reference QRS pattern with short, normalized ECG windows is calculated and the maxima of the correlation are found (R-wave locations). Determination of the RR intervals makes possible calculation of heart rate changes and also heart rate variability (HRV). The algorithm was tested using a simulation in which a noise of an amplitude several times higher than ECG standard deviation levels was added. The proposed algorithm is characterized by high QRS detection accuracy, and high sensitivity and specificity. The algorithm proved to be useful in clinical practice, where it was used to automatically determine HR for ECG signals recorded before and during 58 focal seizures in 56 adult patients with intractable temporal lobe epilepsy.

## Introduction

The average prevalence of active epilepsy is between 0.5 and 1%. So, approximately 65 million people worldwide suffer from this condition [1]. Epilepsy is a disease of the brain defined by any of the following conditions [2]: (a) at least two unprovoked (or reflex) seizures occurring >24 h apart; (b) one unprovoked (or reflex) seizure and a probability of further seizures similar to the general recurrence risk (at least 60%) after two unprovoked seizures, occurring over the next 10 years; (c) diagnosis of an epilepsy syndrome. A seizure is a result of violent bioelectric discharges in neurons which activate specific structural and functional circuits in the brain and disrupt their functioning. During a seizure and shortly thereafter significant changes in heart rhythm may occur [3, 4].

One of the diagnostic parameters used in the study of epilepsy are heart rate changes. This is an important medical problem described in many papers [5–7]. Unfortunately, due to significant physiological abnormalities and artifacts associated with muscle contractions, and movements of a patient’s body during an epileptic seizure, it is very difficult to register clean ECG signals [8]. Electrophysiological recordings (including ECG) registered during a seizure are often distorted with signals of amplitudes as much as several times higher than useable signals. In the article by Eggleston et al. [9] a review of 34 articles that reported the prevalence of ictal tachycardia in patients with epilepsy is conducted. The authors report the occurrence of significant increases in heart rate associated with ictal events in a large proportion of patients with epilepsy (82%). Jansen et al. analysed the ECG signals in the time and frequency domains for 80 seizures [7] before and after the onset of epileptic seizures on EEG. The algorithm of Leutmezer et al. was used to find the temporal relationship between the change in heart rate and seizure onset. Preictal heart rate changes were observed in 70% of the partial seizures. Kato et al. showed that HR abruptly increased in all 29 right seizures and in 42 of 48 left seizures [10]. Onset time of HR increase in relation to ictal EEG onset was significantly earlier in right seizures than in left seizures. Time of maximum HR was also significantly earlier in right seizures than in left seizures. In the article [11] the authors used a Multivariate Statistical Process Control (MSPC) to analyze the HRV. The results produced accurate predictions (91%) for epileptic seizures. Seizure related cardiac arrhythmias are also frequently reported and have been implicated as potential pathomechanisms of Sudden Unexpected Death in Epilepsy (SUDEP) [12]. In particular, postictal arrhythmias, including asystole, AV block and the less prevalent AF and VF, usually occur after a convulsive seizure and are frequently associated with SUDEP. This study aimed to develop a comprehensive system that would enable the measurement of heart rate (HR) for strongly disturbed ECG signals registered during epileptic seizures.

In the system designed by us, the following preprocessing methods were used: IIR filtering and normalization due to skewness and standard deviation. It was assumed that it was possible to create a reference QRS complex for each ECG recording. In the next stage, cross-correlation of short, normalized windows of ECG signals from the reference QRS complex was performed. Then, the correlation maxima were investigated. As a result, a complex algorithm was designed to detect R-waves, to determine RR interval and consequently, heart rate changes. The algorithm was tested with the use of simulations, where noise of an amplitude several times higher than the ECG signal standard deviation was added. It was found that the algorithm had high detection accuracy, and high levels of sensitivity and specificity. The algorithm was also tested in clinical practice, where it was used to automatically determine HR with ECG signals recorded with 58 patients prior to and during epileptic seizures.

## Methods of RR interval detection

**Fig. 1:**
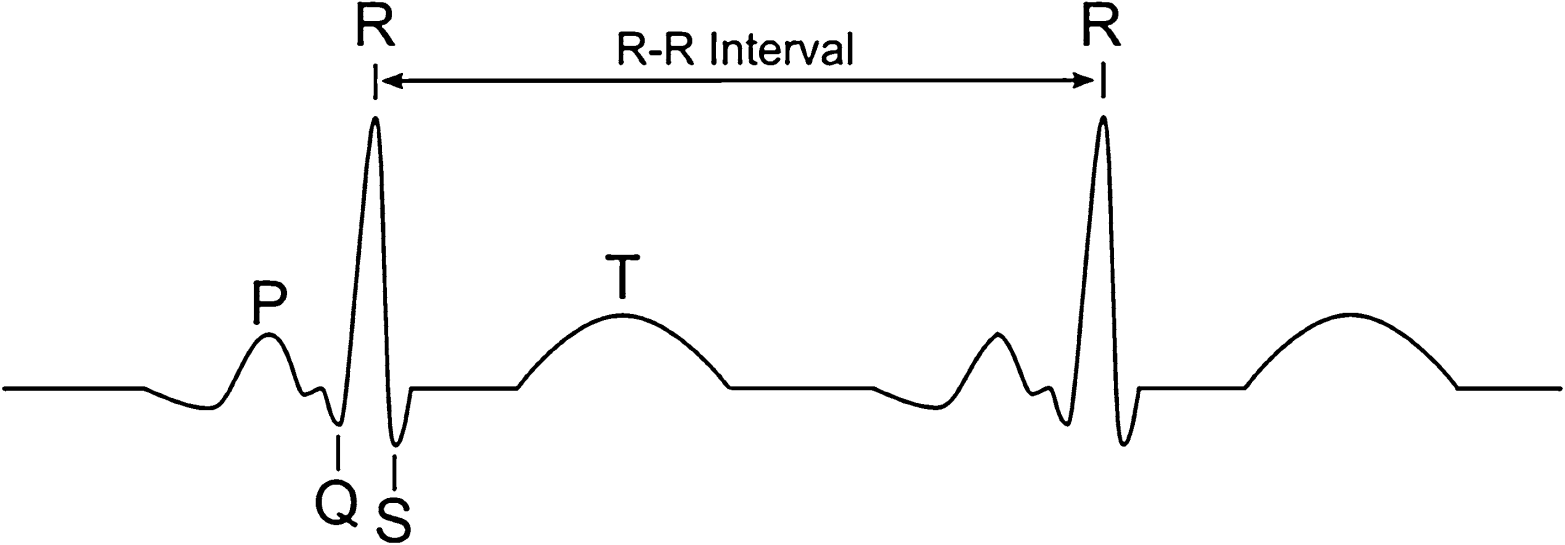
A typical ECG waveform with *P, Q, R, S* and *T* waves

RR interval measurement is enabled via the localization of R-waves in the ECG recording. A typical ECG waveform with P, Q, R, S and T waves is presented in Fig. 1. The length of the PQ segment is usually constant for a patient, i.e. 100–180 ms, the PR section has a length of 120–200 ms, QRS <120 ms, and QT interval <400 ms, while the RR interval is 600–1500 ms.

For typical short ECG recordings, the signals are of very good quality and contain a negligible amount of noise and artifacts. Accordingly, the determination of the RR interval is not difficult. In such a case, a whole range of algorithms for automatic detection of QRS complexes and RR interval determination are known. Pan & Tompkins algorithm [13] is one of the best known and a reference point for many researchers. Generally, signal analyses in time and frequency domains are commonly used. An algorithm which uses a wavelet to detect the QRS complex is proposed in [14]. This algorithm was tested using the MIT-BIH Arrhythmia Database, Third Edition, (May, 1997). The proposed algorithm is characterized by a good detection accuracy. However, as the author himself mentioned, in many cases it is not sufficiently robust toward noise. A method of detecting QRS complexes using Matching Pursuit is proposed in [15]. The authors tested the algorithm on a group of 20 people. It is characterized by a high level of detection accuracy, i.e. 95.3%. Autocorrelation and discrete cosine transform (DCT) coefficients are used for the detection and localization of QRS in [16]. This is an approach which includes impulsive noise suppression and background normalization of digitized electrocardiogram signals, using mathematical morphological operators that incorporate the shape information for a signal [17]. A filter which detects artifacts by keeping track of the statistical properties of the RR-series at three timescales: one global and two local is presented in [18]. The resulting filter shows good flexibility and adaptability to the changing statistical properties of the signal. However most often, the largest signal values (local maxima) are used to detect R-waves [19, 20].

## Materials

Scalp video EEG with simultaneous ECG recordings (vEEG) was performed in patients with refractory temporal epilepsy for long-term monitoring of epileptic seizures during presurgical evaluation. In each case, the ECG activity was recorded with electrodes placed on the chest (*V1–V2* derivation). In 56 patients who underwent epilepsy surgery in the years 2005–2015, ictal vEEG recordings provided localization information and identification of temporal seizure types as well as ECG recordings. After temporal lobe surgery, 43 patients (77%) were completely seizure-free or almost seizure-free (only auras)—Engel class 1 according to the Engel scale [21]—for at least 12 months.

Fifty-eight seizures were recorded on the vEEG with simultaneous ECG in 56 patients (27 men). The temporal ictal onset zone was localized based on seizure ictal semiology and ictal patterns. Using the Wada test, it was found that in 22 patients the seizures started in the hemisphere dominant for language functions, and in the remaining 34 in the hemisphere non-dominant for language functions. 32 bio-signals were registered (1 ECG channel and 31 EEG channels) using an EEG DigiTrack amplifier. The ECG signal was recorded with the sampling frequency *f*_*s*_ = 250 Hz. For EEG registration, cup-shaped electrodes were used and for ECG registration—precordial disposable electrodes located at *V1* and *V2* positions. During signal registration, patients were either sitting or lying, but they could freely move their heads and limbs. The registration time was from several minutes to several hours.

## Algorithm for RR interval detection

The research assumption was that the system had to be robust toward disturbances and artifacts that occur during epileptic seizures. This requires effective preprocessing methods, in particular filtration. The type and parameters of the electrodes, the manner of their attachment to the chest and QRS complex morphology (which is individual for each patient) can lead to significant variation in ECG recordings. It was assumed that the QRS complex morphology for a patient does not change either before epileptic seizures or during the seizures. Additionally, it was assumed that the ECG signal registered before the seizure is free from disturbances and that R-wave detection is relatively easy. Our algorithm should give the average heart rate in short time intervals [22]. The algorithm is designed to work in off-line mode.

**Fig. 2:**
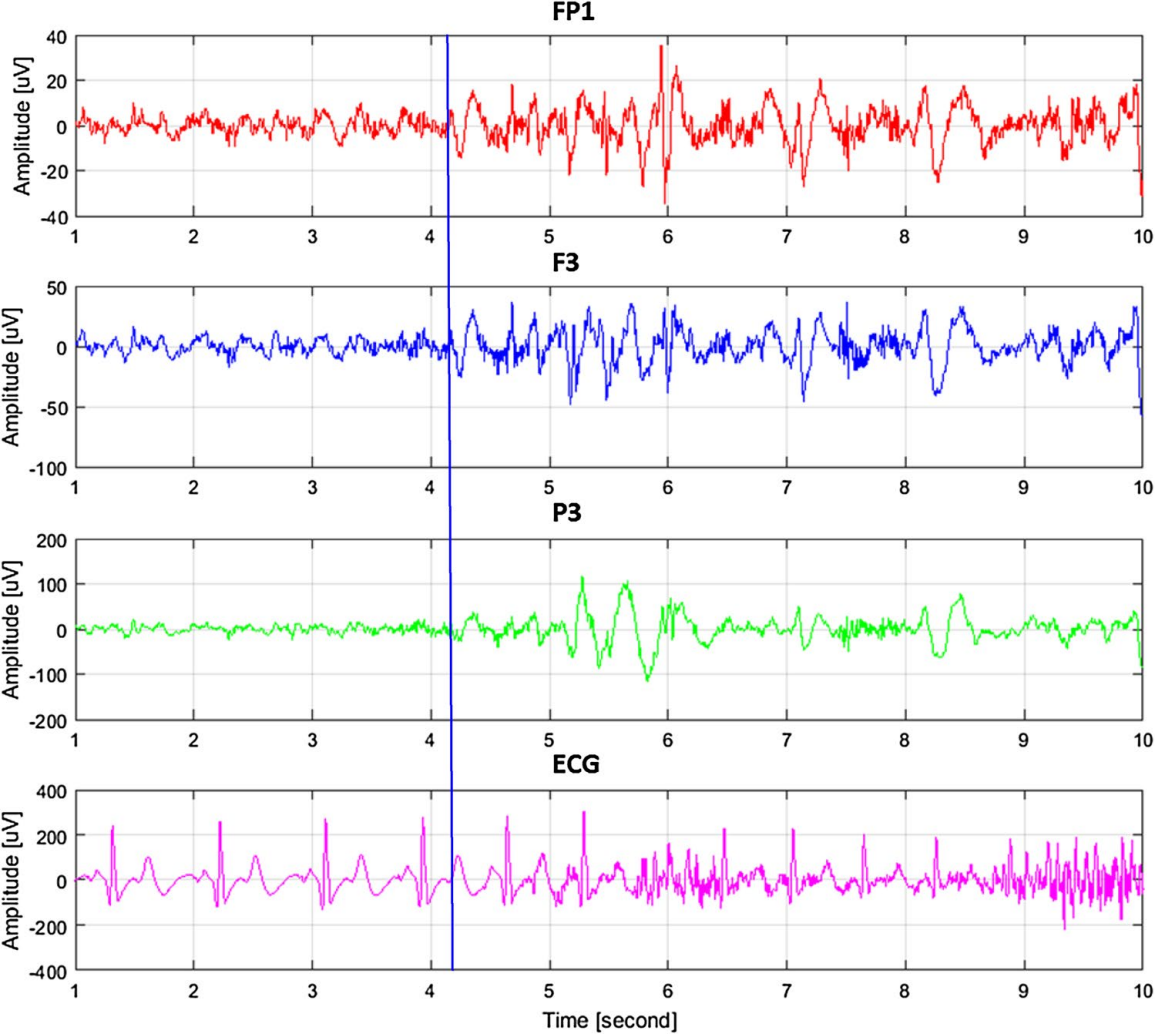
A typical ECG and EEG signals immediately prior to and during epileptic seizure—from top: EEG (FP1, F3, P3) and ECG

Examples of ECG and EEG waveforms recorded by us immediately prior to and after the beginning of an epileptic seizure (vertical line in 4.2 s) are shown in Fig. 2. After the beginning of an epileptic seizure muscle activity that causes artifacts can be observed. These artifacts are also visible in the recorded ECG signal. Synchronization of EEG channels characteristic for epileptic seizures and significant ECG signal distortion are clearly visible.

It should be emphasized that the ECG signals, during epileptic seizures, are often affected by the biological and environmental factors listed below and these need to be considered before detecting actual QRS complexes [23]:

- Interferences from the power network 50/60 ± 0.2 Hz,
- Temporary loss of skin-electrode contact,
- Electrode shifts on the skin,
- Electromyographic (EMG) noise (electrical activity due to muscle contractions lasting around 50 ms, in the band 0–10 kHz),
- Movements from respiration at frequencies drifting between 0.15 and 0.3 Hz,
- Artifacts generated by signal processing hardware such as signal saturation,
- Noise generated by other medical equipment present in the patient care environment at frequencies between 100 and 1 MHz.

**Fig. 3:**
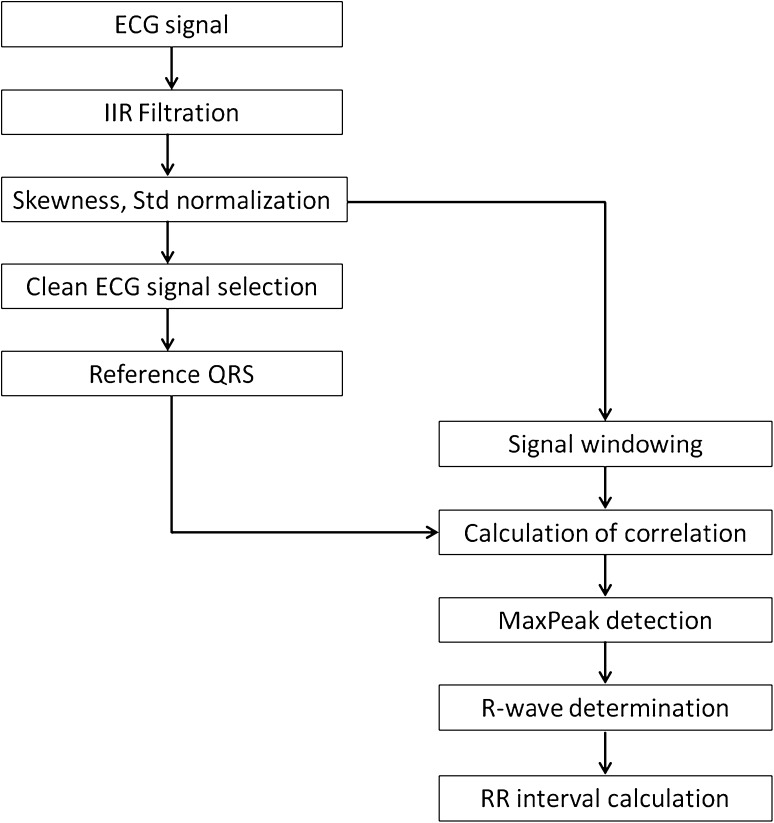
Diagram of the system for determining the RR intervals

The system for determining the RR intervals, proposed by us, is presented in Fig. 3. The first element of the system is IIR filtration (*IIR Filtration*). Its purpose is to eliminate interfering signals from the power network (50 Hz) and low-frequency drifts. Two Butterworth filters were used: band-pass (4–80 Hz) and band-stop (48–52 Hz). Next, the signal amplitudes were normalized (*Skewness, Std normalization*). If the skewness is less than zero the signal polarity is reversed. The result is a normalized ECG signal (standard deviation equal one) with R-waves of a positive polarity.

Then, the first 30-s part of the ECG signal for a patient is selected (assumed undistorted)—(*Clean ECG signal selection*). This fragment of the ECG signal is used to determine the reference QRS complex. To calculate this, local signal maxima are used for R-wave detection. In our algorithm, the minimum value of amplitude was 2.5 and the minimum time interval between R “peaks” (RR interval) was 80 samples (0.32 s).

**Fig. 4:**
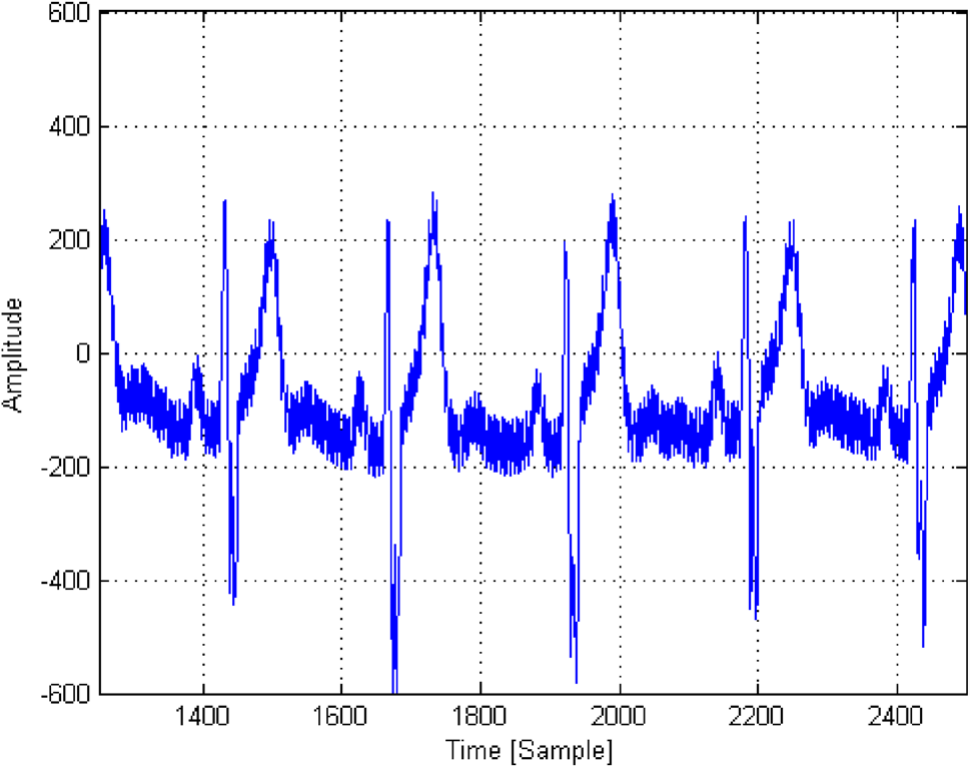
Raw ECG signal

**Fig. 5:**
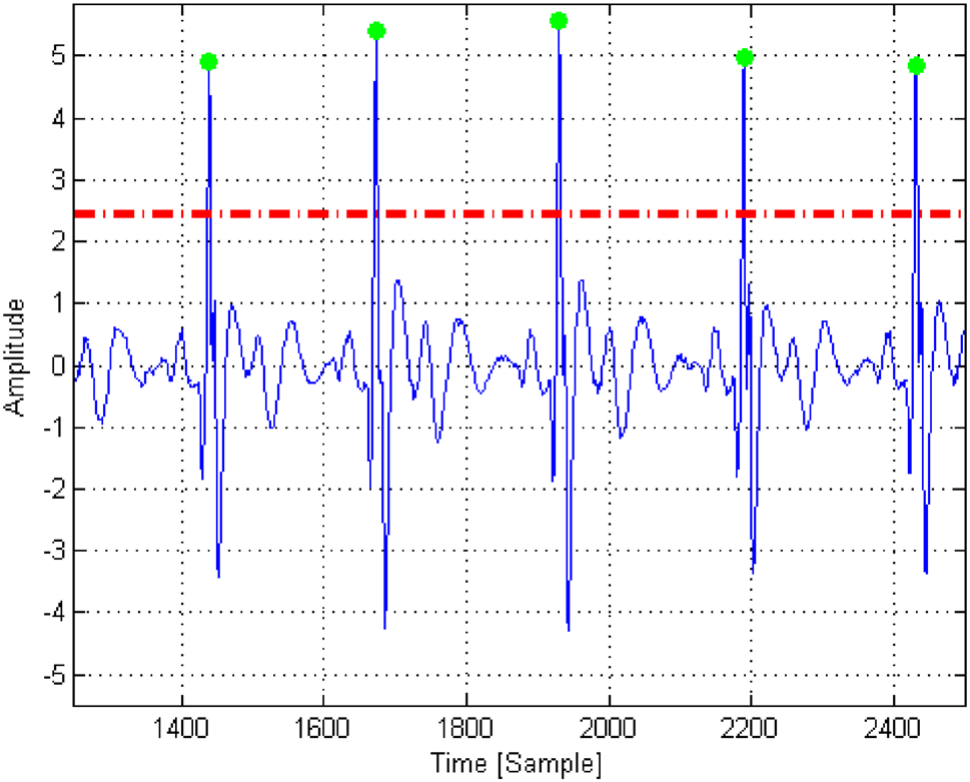
A registered ECG signal after band-pass (4–80 Hz) and band-stop (48–52 Hz) filtering and normalization

A raw ECG signal is presented in Fig. 4. Figure 5 presents the signal from Fig. 4, filtered using bandpass (4–80 Hz) and band-stop (48–52 Hz) filters, after normalization on the basis of standard deviation and skewness. The red line indicates the threshold level value of 2.5 units used to detect of local maxima. The green dots indicate the results of the automatic detection of local maxima, associated with the R-waves in the QRS complex.

**Fig. 6:**
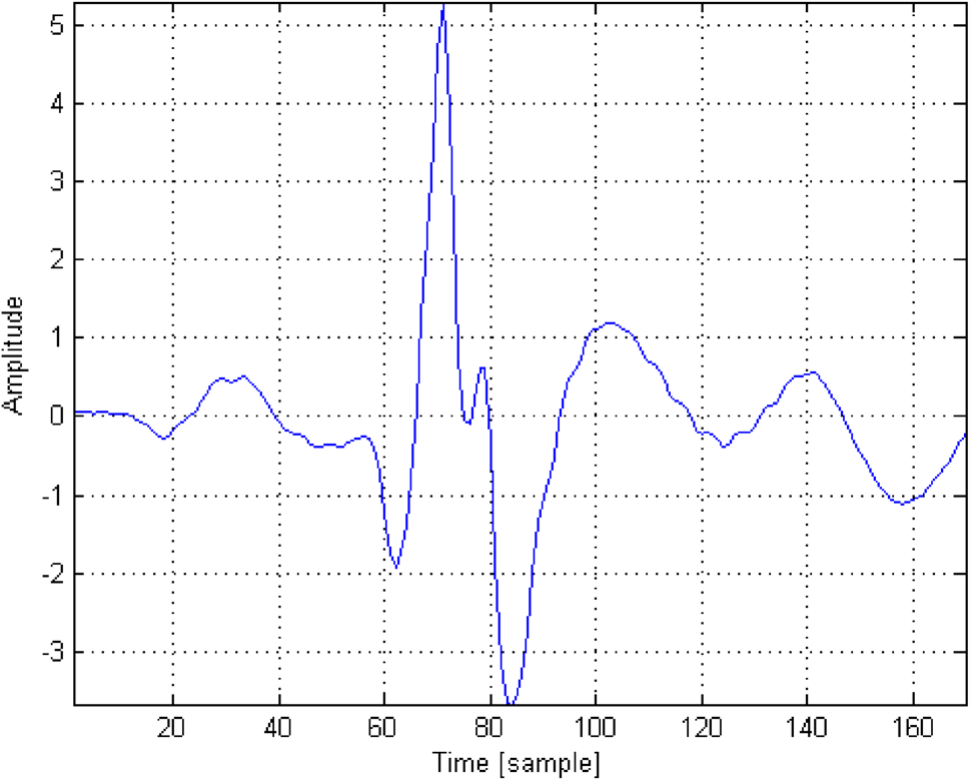
A sample reference QRS complex (${{\Psi }}_{QRS}$)

Then, the ECG signal fragments containing QRS complexes were averaged. The averaged ECG fragments contained a section of 70 samples (0.28 s) before the R-wave and 100 samples (0.4 s) after the R-wave (*Reference QRS*). A reference QRS complex (labeled ${{\Psi }}_{QRS}$) for a specific ECG waveform calculated in this manner is presented in Fig. 6. Based on the authors’ assumption, the QRS morphology remains unchanged for a patient. Such an approach allows the identification of a specific pattern which best reflects the QRS nature for each person.

In the following step, the ${{\Psi }}_{QRS}$ time window (171 samples) was slid along the ECG signal with a large overlap of 170 samples (*Signal windowing*). For each ${{\Psi }}_{QRS}$ window position, the correlation of ECG signal covered by the window with the ${{\Psi }}_{QRS}$ signal was determined (*Calculation of correlation*). The Pearson correlation coefficient was used to calculate this [24]. Thus, a *C* waveform is created, termed simply the correlation. In the next step, the *C* correlation was divided into ${Cw}_{n}$ windows of a duration of 500 samples (2 s) overlapping in 150 samples (0.6 s). For each ${Cw}_{n}$ window, the following were performed (*MaxPeak detection*):

1. Normalization of signal $C{w_n}=C{w_n}/\max { }(C{w_n})$
2. Determination of local maxima for normalized ${Cw}_{n}$ windows.

Determination of the local maxima required a minimum threshold level of 0.7 units and a minimum time distance between the peaks of 80 samples (0.32 s). The determined maxima corresponded to the location of R-waves in the ECG signal. As the windows overlapped, the danger of duplication of R-waves occurred. Therefore, in a further step R-wave duplicates (*R-wave determination*) were eliminated. The last step was to determine the RR intervals by calculating the distance between R waves (measured in number of samples). The instantaneous number of heart beats per minute HR was calculated from the formula:

1 $$HR=\frac{{60{f_s}}}{{{I_{RR}}}}[\text{bpm}]$$

where *f*_*s*_ sampling rate, *I*_*RR*_ the instantaneous value of the interval between R-waves, 60 conversion of seconds to minutes. As a result, an automated system was created which allows the calculation of heart beats per minute for any point of time.

## Results

**Fig. 7:**
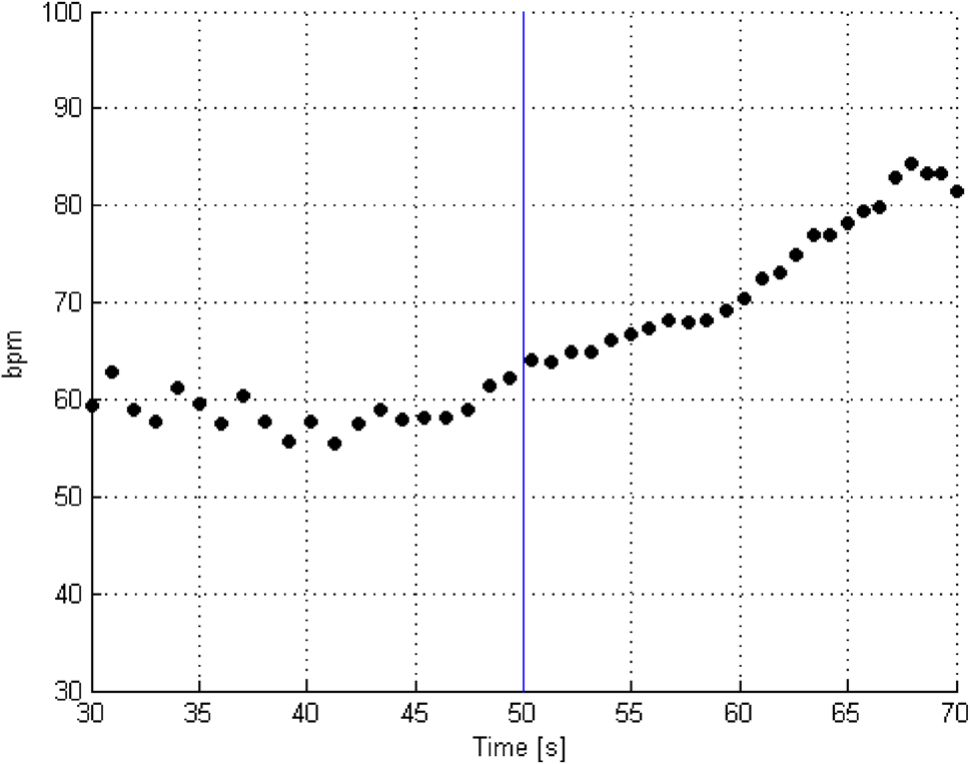
A typical changes of HR value before and during epileptic seizure (the *vertical blue line* shows the beginning of an epileptic seizure)

A typical changes of HR value before and during epileptic seizure are shown in Fig. 7. The onset of the clinical seizure (as determined by a physician) occurs at 50 s. There is an increase in heart rate immediately prior to and after the clinical seizure. This is consistent with medical knowledge [4, 25].

**Fig. 8:**
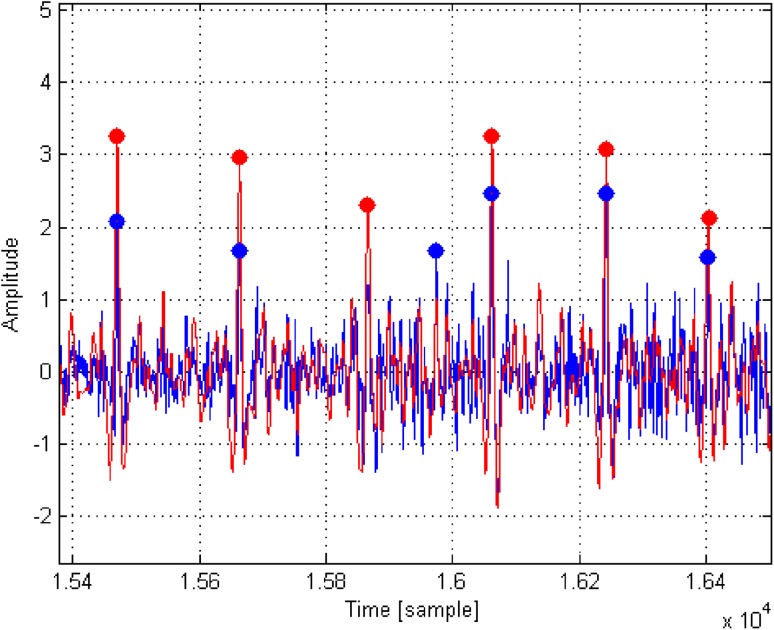
Detection of HR (location of R-waves)

Figure 8 presents an example of the detection of heart rate (location of R-waves). The blue color shows a fragment of noisy ECG signal with artifacts, the red color—the *C* correlation signal. Points represent maxima determined for the ECG signal (blue color) and for the *C* correlation (red color).

**Fig. 9:**
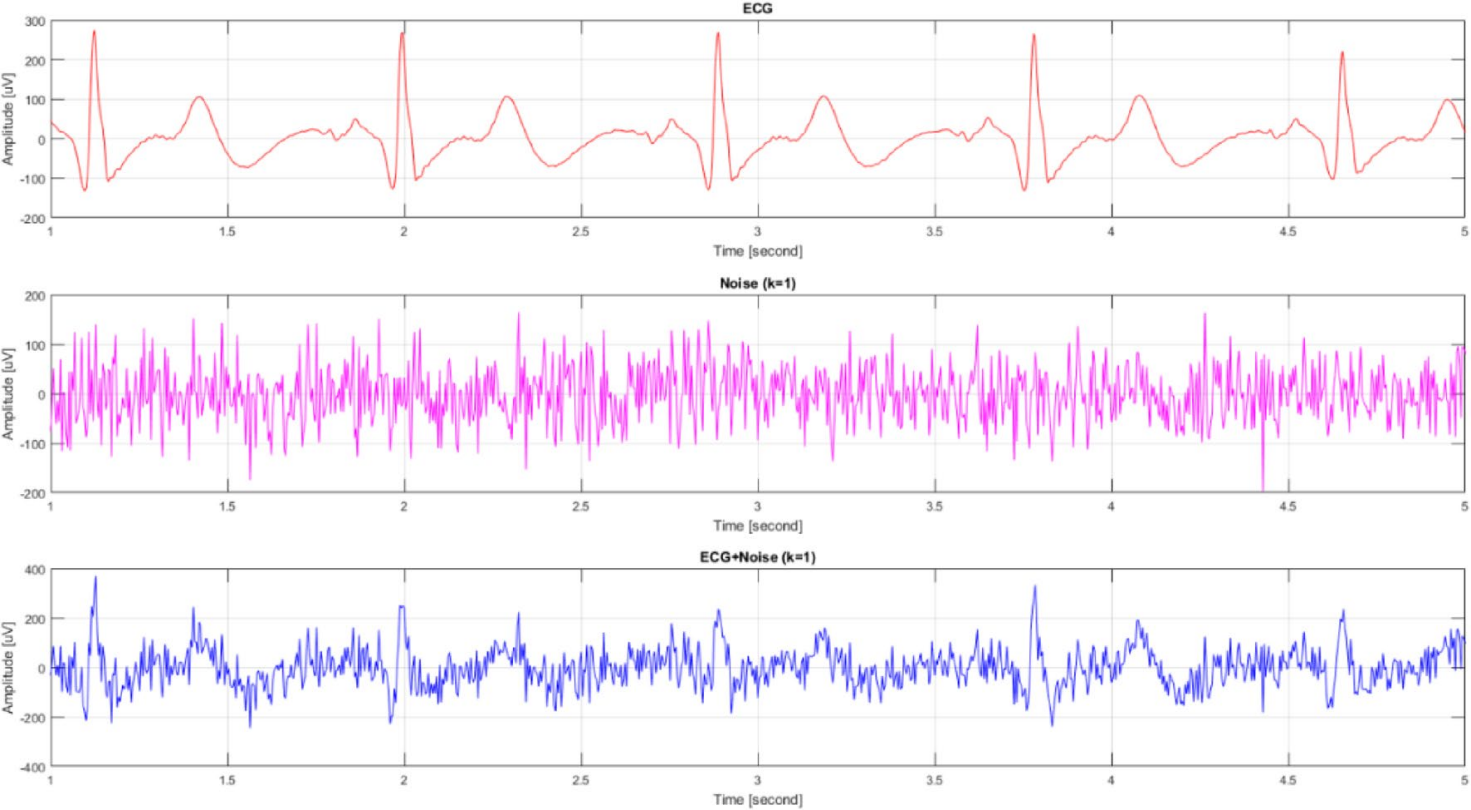
ECG waveform, a noise signal of strength *k* and the ECG signal with added noise

An incorrectly detected R-wave can be observed in the ECG signal (range 1.58–1.60). This false R-wave is ignored by our algorithm—a correct result for the analysis is achieved. However, there were cases in which our algorithm did not allow the accurate detection of the R-waves. In order to evaluate the performance of the developed system and compare it with a simple method of direct signal maxima detection, the influence of added noise on the R-wave detection accuracy was examined. The idea was to achieve the effect of the occurrence of muscle artifacts (EMG) [26] and other disturbances. For each user a 30-s good quality ECG signal was selected and then a noise was added to it. The noise level was controlled by a *k* coefficient. This coefficient can be interpreted as a measure of the ratio between the amplitude of ECG signal, and the amplitude of the artifacts. Table 1 presents the relationships between standard deviation (*std*) and maximum values (*max*) for the ECG signals. Figure 9 shows an example of ECG waveform, a noise signal of strength *k* = 1 and the ECG signal with added noise (*ECG* + *Noise*).

**Table 1:** Parameters of ECG signal with added noise

	Noise		ECG + noise	
	Std (uV)	Max (uV)	Std (uV)	Max (uV)
*k = 0 (no noise)*	–	–	58.0	306.9
*k = 1*	58.9	215.9	82.6	376.6
*k = 2*	11.9	431.9	131.3	490.5
*k = 3*	176.8	647.8	186.0	653.2
*k = 4*	235.8	863.8	242.7	866.1
*k = 5*	294.7	1079.7	300.3	1079.0
*k = 6*	353.6	1295.7	358.3	1291.9
*k = 7*	412.6	1511.6	416.6	1504.8

In this way, it was possible to compare the location of R-waves obtained by determining the signal maxima for raw ECG signals in a direct manner with the results of our proposed method for the determination of the maxima. Tables 2, 3 and 4 show in sequence: the accuracy, specificity and sensitivity of R-wave detection for different SNR for noisy ECG signals. The results were calculated for 58 focal seizures in 56 adult patients with intractable temporal lobe epilepsy.

**Table 2:** Accuracy of R wave detection

	*K*							
	0	1	2	3	4	5	6	7
*C*	0.99	0.98	0.92	0.89	0.72	0.61	0.44	0.39
ECG	0.99	0.8	0.5	0.25	0.20	0.17	0.12	0.14

**Table 3:** Specificity of R wave detection

	*k*							
	0	1	2	3	4	5	6	7
*C*	0.98	0.97	0.86	0.83	0.68	0.58	0.44	0.42
ECG	0.98	0.99	0.66	0.33	0.27	0.23	0.17	0.19

**Table 4:** Sensitivity of R wave detection

	*k*							
	0	1	2	3	4	5	6	7
*C*	1	1	0.99	0.96	0.75	0.65	0.44	0.37
ECG	1	0.67	0.4	0.19	0.16	0.13	0.1	0.1

## Discussion

During the calculations, the accuracy of the detection of the R-waves in noisy ECG signals was considered correct if the original R-wave appeared at a distance of no more than three samples (0.012 s). Otherwise, it was considered that the R-wave was not properly detected. For this assumption, the maximum error for measured heart rate (expressed in beats per minute deviation) is presented in Table 5. The maximum error increases with the number of beats per minute and reaches 3.29 beats for 130 bpm.

**Table 5:** The maximum error of measured heart rate

Pulse (bpm)	60	70	80	90	100	110	120	130
Error (bpm)	0.71	0.96	1.25	1.59	1.96	2.36	2.81	3.29

The results presented in Tables 2, 3 and 4 clearly indicate that the developed system has a much better accuracy, specificity and sensitivity of R-wave detection than the direct method of detecting local maxima in ECG signals. For example, after adding a noise to an ECG with an amplitude three times greater than the standard deviation of the ECG signal, very good results were obtained––that is, a detection accuracy of 0.89, specificity 0.83 and detection sensitivity 0.96 compared to an accuracy of 0.25, specificity 0.33 and detection sensitivity 0.19 for finding maxima directly in the ECG signal.

**Fig. 10:**
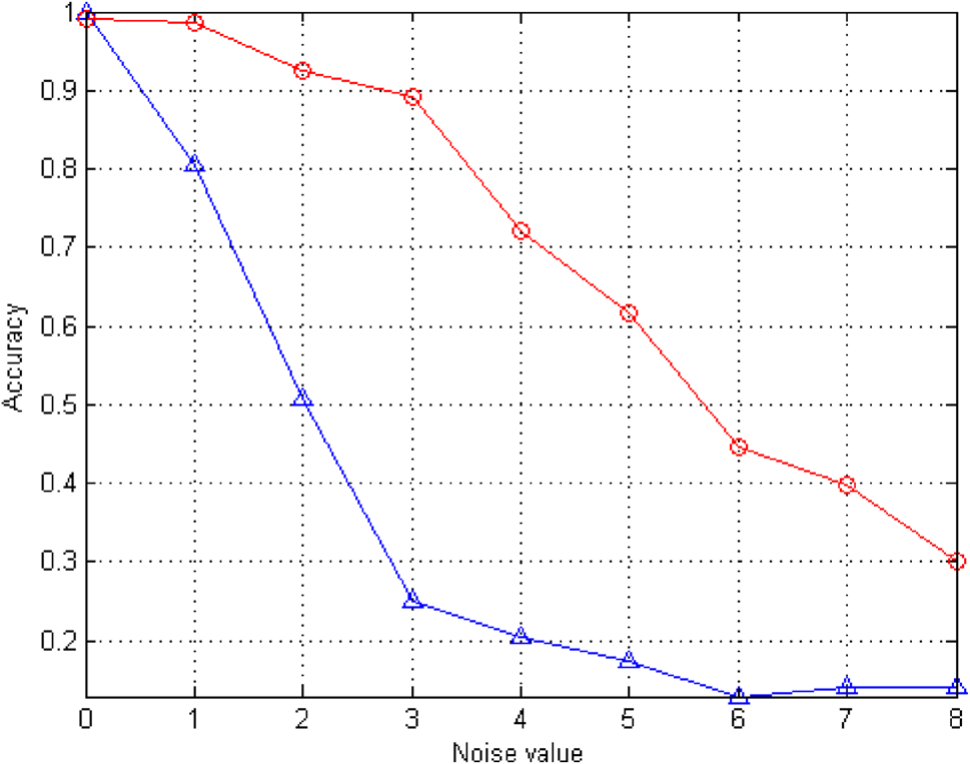
R-wave detection accuracy, *blue color*—the direct method, *red color*—the correlation method

The comparison of the accuracy of R-waves detection for both methods is given in Fig. 10. The blue color shows the accuracy of detection for the direct method, while the red color depicts the accuracy with the use of the correlation method.

**Fig. 11:**
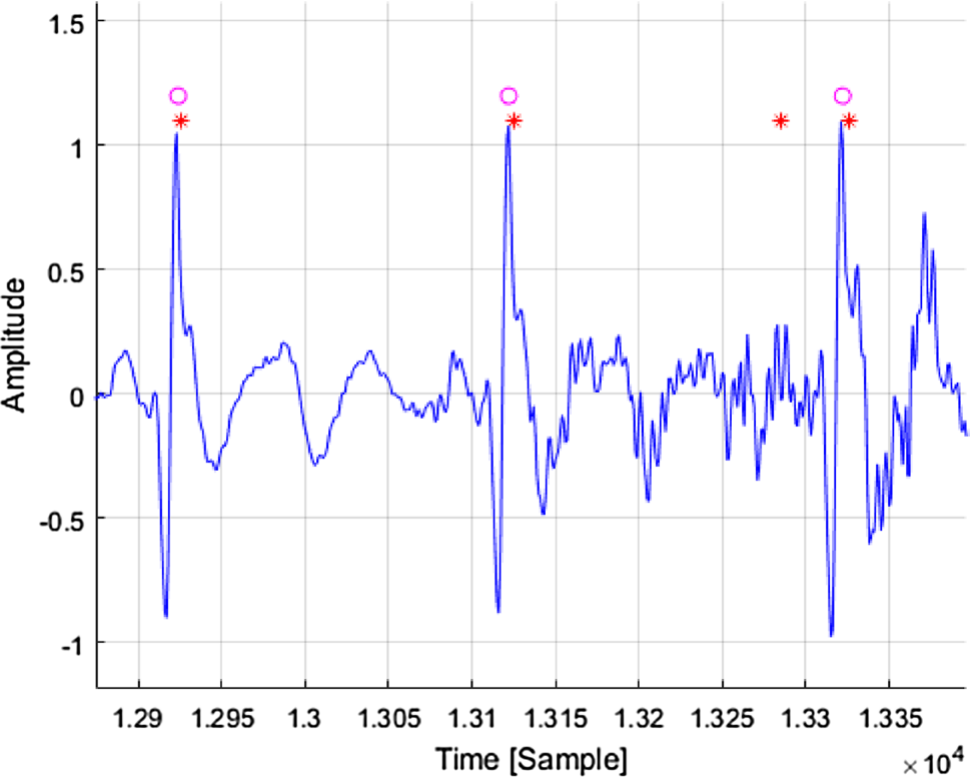
The results of the R-wave detection with the use of Pan & Tompkins (*asterisk*) and our (*open circle*) algorithms for the real ECG signal disturbed by natural muscle artifacts

It is difficult to directly compare the created system with systems described in other studies, since the majority of examples of the described algorithm have been implemented to detect R-waves in ECG signals of good quality. The accuracy of R-wave detection for good quality ECG signal scan be as high as 83–96% [15, 27, 28]. Nevertheless we compared our solution with a known Pan & Tompkins algorithm [13]. Both our and Pan & Tompkins algorithms function properly in the event of undistorted ECG signal. Distinct differences were seen only during the R-wave detection for ECG signal containing artifacts. Figure 11 illustrates an exemplary fragment of a real ECG signal recorded at the start of epileptic seizure. In case of Pan & Tompkins algorithm a redundant R wave detection can be observed. This is due to the imposition of muscle artifacts (EMG) on the ECG signal. Our algorithm does not revealed at this point a redundant R-wave. This situation often repeated.

**Fig. 12:**
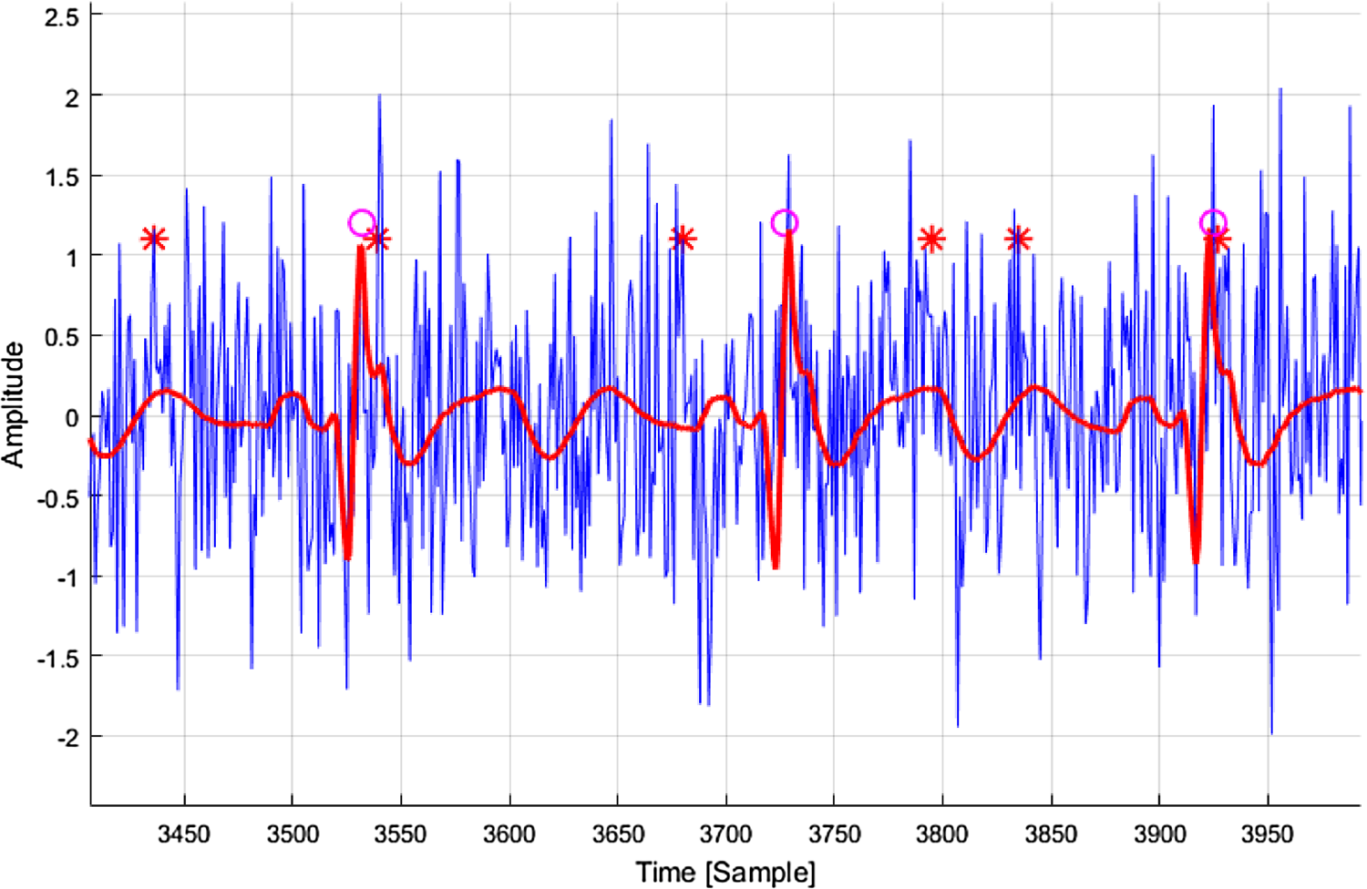
The results of the R-wave detection with the use of Pan & Tompkins (*asterisk*) and our (*open circle*) algorithms

In Fig. 12 the comparison of the same algorithms for the real ECG signal with addition of EMG artifacts, simulated in the form of noise with parameter *k* = 1, is presented. Red color indicates undisturbed ECG signal, blue—the corresponding one with added noise. It is easy to observe redundant detections of R waves in the case of the Pan & Tompkins algorithm.

The proposed system was tested in offline mode, but it can be easily implemented on-line. However, a limitation of the system is the assumption that the first part of the ECG signal, appearing before the seizure, does not contain significant artifacts, which may affect the determination of the reference QRS complex.

## Conclusion

The system developed for the detection of heart rate during epileptic seizures is characterized by high detection accuracy, high specificity and high sensitivity. It allows the detection of R-waves in signals with a high level of noise and artifacts. After verification in clinical practice, it was confirmed that it greatly facilitates diagnosis of heartbeats before, after and during epileptic seizures. The system can also be used as part of the diagnosis of heart rate variability in other heart disorders, not only epilepsy.
